# A New Glabrous Gene (*csgl3*) Identified in Trichome Development in Cucumber (*Cucumis sativus* L.)

**DOI:** 10.1371/journal.pone.0148422

**Published:** 2016-02-04

**Authors:** Jin-Ying Cui, Han Miao, Li-Hong Ding, Todd C. Wehner, Pan-Na Liu, Ye Wang, Sheng-Ping Zhang, Xing-Fang Gu

**Affiliations:** 1 Institute of Vegetables and Flowers, Chinese Academy of Agricultural Sciences, Beijing, China; 2 Department of Horticultural Science, North Carolina State University, Raleigh, NC, United States of America; National Institute of Plant Genome Research (NIPGR), INDIA

## Abstract

Spines or trichomes on the fruit of cucumbers enhance their commercial value in China. In addition, glabrous mutants exhibit resistance to aphids and therefore their use by growers can reduce pesticide residues. Previous studies have reported two glabrous mutant plants containing the genes, *csgl1* and *csgl2*. In the present study, a new glabrous mutant, NCG157, was identified showing a gene interaction effect with *csgl1* and *csgl2*. This mutant showed the glabrous character on stems, leaves, tendrils, receptacles and ovaries, and there were no spines or tumors on the fruit surface. Inheritance analysis showed that a single recessive gene, named *csgl3*, determined the glabrous trait. An F_2_ population derived from the cross of two inbred lines 9930 (a fresh market type from Northern China that exhibits trichomes) and NCG157 (an American processing type with glabrous surfaces) was used for genetic mapping of the *csgl3* gene. By combining bulked segregant analysis (BAS) with molecular markers, 18 markers, including two simple sequence repeats (SSR), nine insertion deletions (InDel) and seven derived cleaved amplified polymorphism sequences (dCAPs), were identified to link to the *csgl3* gene. All of the linked markers were used as anchor loci to locate the *csgl3* gene on cucumber chromosome 6. The *csgl3* gene was mapped between the dCAPs markers dCAPs-21 and dCAPs-19, at genetic distances of 0.05 cM and 0.15 cM, respectively. The physical distance of this region was 19.6 kb. Three markers, InDel-19, dCAPs-2 and dCAPs-11, co-segregated with *csgl3*. There were two candidate genes in the region, *Csa6M514860* and *Csa6M514870*. Quantitative real-time PCR showed that the expression of *Csa6M514870* was higher in the tissues of 9930 than that of NCG157, and this was consistent with their phenotypic characters. *Csa6M514870* is therefore postulated to be the candidate gene for the development of trichomes in cucumber. This study will facilitate marker-assisted selection (MAS) of the smooth plant trait in cucumber breeding and provide for future cloning of *csgl3*.

## Introduction

Trichomes are hair-like structures that are widely present on the surface of the aerial organs of plants, including stems, leaves, tendrils, calices and ovaries [1]. As a first line of defence, trichomes function in protecting plants against excessive transpiration, and damage by insects, pathogens, herbivores, UV irradiation, and low temperature [2–4]. In addition, trichomes may help plants attract pollinators and disperse seeds [5].

A histological analysis by Guan et al. [6] showed that the leaf trichomes and fruit spines of cucumber have a similar shape and structure, both being multicellular and non-glandular, unlike the single-celled, branched trichomes in *Arabidopsis* or the multiple types of trichomes in tomato [7–9]. There are two types in cucumber: type I trichomes are tiny, with a three-to-five cell base topped with a four-to-eight cell head, and these have been shown to be involved in cuticle formation; type II trichomes, the dominant type, are larger, with a conical shape, and are non-glandular and branchless [10,11].

Cao et al. [12] isolated a spontaneous mutant (*csgl1*) from a north China-type cucumber cultivar ‘Daqingba’. All aerial parts of this cultivar were glabrous, including leaves, stems, tendrils, floral organs and fruits. A genetic analysis showed that *csgl1* was recessive epistatic effect to the *tubercule gene* (*Tu*), and was located on the same side of SRAP markers ME4EM3 and ME23OD15 [6]. Zhang et al. [13] used the sequence-related amplified polymorphism technique (SRAP) to locate two dominant markers, ME6EM5 and ME23OD15, linked to *csgl1*, at distances of 3.6 cM and 12.9 cM, respectively. A further two markers were detected, one of which, ME4EM3, was linked with a distance of 3.2 cM. The gene of *csgl1* was fine mapped to a region with a physical distance of 79.7 kb enclosing 13 candidate genes [14]. Two candidate genes, *CsMYB6* and *CsGA20ox1*, possibly involved in the formation of cucumber trichomes were characterized, and *CsMYB6* has been shown to have a role in the abiotic stress responses of plants and is strongly expressed in trichomes and fruit spines [14].

Another cucumber mutant, *csgl2*, was characterized by Yang et al. [15] from a cucumber mutant that exhibits few trichomes or tubercules on the tendrils, calices, ovaries and fruit, but glabrous stems and leaves. The glabrous trait was determined to be due to a single nuclear gene which is recessive to the wild type. Markers SSR10522 and SSR132751 were linked to *csgl2* on chromosome 2 with genetic distances of 0.6 cM and 3.8 cM, respectively.

Several researchers have reported cloning trichome-related genes. Mutants of the *TRANSPARENT TESTA GLABRA1* (*TTG1*) locus in *Arabidopsis* showed none of the trichomes that are normally produced by meristematic cells of the wild type. Mutant alleles of the cloned gene, *ttg1*, produced either truncations or single amino acid changes in the TTH1 protein [16]. The trichomes and root hairs of *Arabidopsis* were reported to be under the putative control of a number of related transcription factors. Kirika et al. [17] reported that over expression of some of these factors by a new regulator, *ENHANCER OF TRY AND CPC 1*, caused a reduction in trichome formation and excessive root hair production. Yang et al. [18] cloned the *Woolly* gene (*Wo*) from tomato, which was essential for trichome formation. Suppression of this gene by RNA interference (RNAi) decreased trichome formation.

Formation mechanism of trichomes was a complex process with many genes regulation. Several trichome-related genes have been studied in *Arabidopsis thaliana* [2–4], such as *GLABRA3* (*GL3*), *ENHANCER OF GLABRA3* (*EGL3*), *GLABRA1* (*GL1*) and so on [11]. In cucumber, two transcription factors related to trichomes, *csgl1* and *csgl2*, have been found [14,15]. This study aimed to map a third putative mutant gene, *csgl3*, in the glabrous mutant NCG157, and to examine the expression analysis of the candidate genes for this mutant.

## Materials and Methods

### Plant materials

NCG157, a glabrous mutant, was provided by Prof. Todd C. Wehner, Department of Horticultural Science, North Carolina State University. It is an American processing type cucumber with glabrous surfaces. 9930 is a fresh market type from Northern China that exhibits trichomes. The genome of 9930 has been sequenced [19] and annotated (http://www.icugi.org/cgi-bin/ICuGI/index.cgi). The cross of 9930 (P_1_)×NCG157 (P_2_) was made for fine mapping and exploring the *csgl3* gene.

To determine the possible interactive relationship of the glabrous genes, three glabrous lines, 1945 (containing glabrous gene *csgl1*), NCG042 (containing glabrous gene *csgl2*), NCG157 (putatively containing glabrous gene *csgl3*) were used in this study. Three cross combinations were constructed: 1945×NCG042, 1945×NCG157, NCG042×NCG157.

All plants were grown in a greenhouse under natural sunlight at the Institute of Vegetables and Flowers, Chinese Academy of Agricultural Sciences. The temperature in the greenhouse was 25–30°C during the day and 18–25°C during the night, and 30%–85% relative humidity. All experiments were analyzed using the SAS software program (SAS 9.2).

### Phenotype measurement and scanning electron microscopy (SEM) analysis

All populations were scored twice for the hairy trait at the stage of two true leaves. Scoring was carried out by touch for rough or smooth. If trichomes were detected on a leaf, they were also present on the stem of the same plant.

SEM of cucumber trichomes was performed on the leaves, cotyledons, stems and roots of young plants from 9930 and NCG157 as described by Chen et al. [11].

### DNA extraction and bulked segregant analysis (BSA)

Genomic DNA was extracted from young leaves with the modified CTAB method [20]. A UV spectrophotometer was used to measure DNA purity and concentration, and the final concentration was diluted to 30 ng/μL. Equal amounts of DNA from seven hairy plants and seven glabrous plants, randomly selected from the F_2_ populations, were pooled to construct two DNA bulks for BSA [21].

### Molecular marker development and linkage analysis

A total of 2,112 SSR primers developed by the International Cucurbit Genomics Initiative (ICuGi) [22], were used to screen polymorphisms in the two parents and two bulks. Based on the sequence of the genome between SSR16882 and SSR02460, further screening was carried out with 212 UW SSR marker (obtained from the University of Wisconsin), 42 InDel and 19 dCAPs markers. All primers were synthesized by the Shanghai Sangon Biological Engineering Technology & Services Co., Ltd., Shanghai, China.

The PCR conditions were as described by Zhang et al. [23]. The amplification conditions were as follows: 4 min at 94°C, 35 cycles of 15 s at 94°C, 30 s at 55°C, 30 s at 72°C, and a final extension of 5 min at 72°C. 6.0% non-denatured polyacrylamide gels with 0.5×TBE buffer were used to separate the amplification products at a constant power of 150 V for 55 min. After electrophoresis, the gel was silver-stained [24] and photographed with a digital camera (Sony).

### Mapping and candidate genes annotation

Data from the F_2_ populations were used to map the glabrous gene in NCG157. The phenotypic, SSR, InDel and dCAPs analyses were combined for linkage analysis using the Joinmap 4.0 program with a LOD threshold of 3.0.

### Candidate genes prediction

Potential candidate genes in the target genomic region were identified using Softberry (http://linux1.softberry.com/all.htm) and the Cucumber Genome Database (http://www.icugi.org/cgi-bin/ICuGI/index.cgi). The functional annotations of these genes were acquired either from the databases or from links to the National Center for Biotechnology Information (NCBI) databases. Primers were designed with Primer Premier 6.0 (PREMIER Biosoft, Palo Alto, CA, USA).

### Analysis of candidate genes that were differentially expressed

The cucumber tissues (roots, leaves, stems and fruit skins) from both parents were peeled off with a knife and flash frozen in liquid nitrogen. There were three technical replications and biological repetitions for each parent. In order to correct the expression of target genes, we chose *Actin1* as reference [25]. The Takara kit for total RNA isolation and cDNA synthesis (Takara Biomedical Technology (Beijing) Co., Ltd., Beijing, China), was used for candidate genes analysis. Approximately 100 mg of frozen cucumber tissues were disrupted in liquid nitrogen using a mortar and pestle, and suspended in a mixture of buffers RL and DTT (supplied with the Takara kit). Total RNA extraction was performed according to the manufacturer’s protocol. The RNA pellet was isolated by RNA spin column, and dissolved in 100 μL of RNase free water. To avoid any DNA contamination, samples were treated with DNAse I (5 μL 10×DNase I buffer, 4 μL Recombinant DNase I and 4 μL RNase free water) at 25°C for 15 min. The reaction was stopped by the addition of 350 μL of buffer RWB (supplied with the Takara kit). After allowing time for equilibration and refolding, the RNA concentration and purity was determined both before and after DNA digestion by spectrophotometry and agarose gel electrophoresis.

For cDNA synthesis, 1 μg of total RNA was mixed with 2 μL of 5×PrimeScript RT Master Mix (Takara Biomedical Technology (Beijing) Co., Ltd., Beijing, China), and made to 10 μL with RNase free water. The Reverse Transcription System was used according to the manufacturer’s instructions. Quantitative real-time PCR was performed in a volume of 25 μL. Reaction mixtures contained 1 μL of cDNA, 12.5 μL of SYBR Green Master Mix (Takara Biomedical Technology (Beijing) Co., Ltd., Beijing, China), and 1 μL each of 10 μM primers in a total volume of 25 μL. Negative control PCRs contained 1 μL of RNase free water instead of cDNA. The following amplification conditions were applied: 30 s at 95°C, 45 cycles of 10 s at 95°C, 10 s at 55°C, 15 s at 72°C, and 15 s at 65°C.

## Results

### Morphological characterization analysis

**Fig 1:**
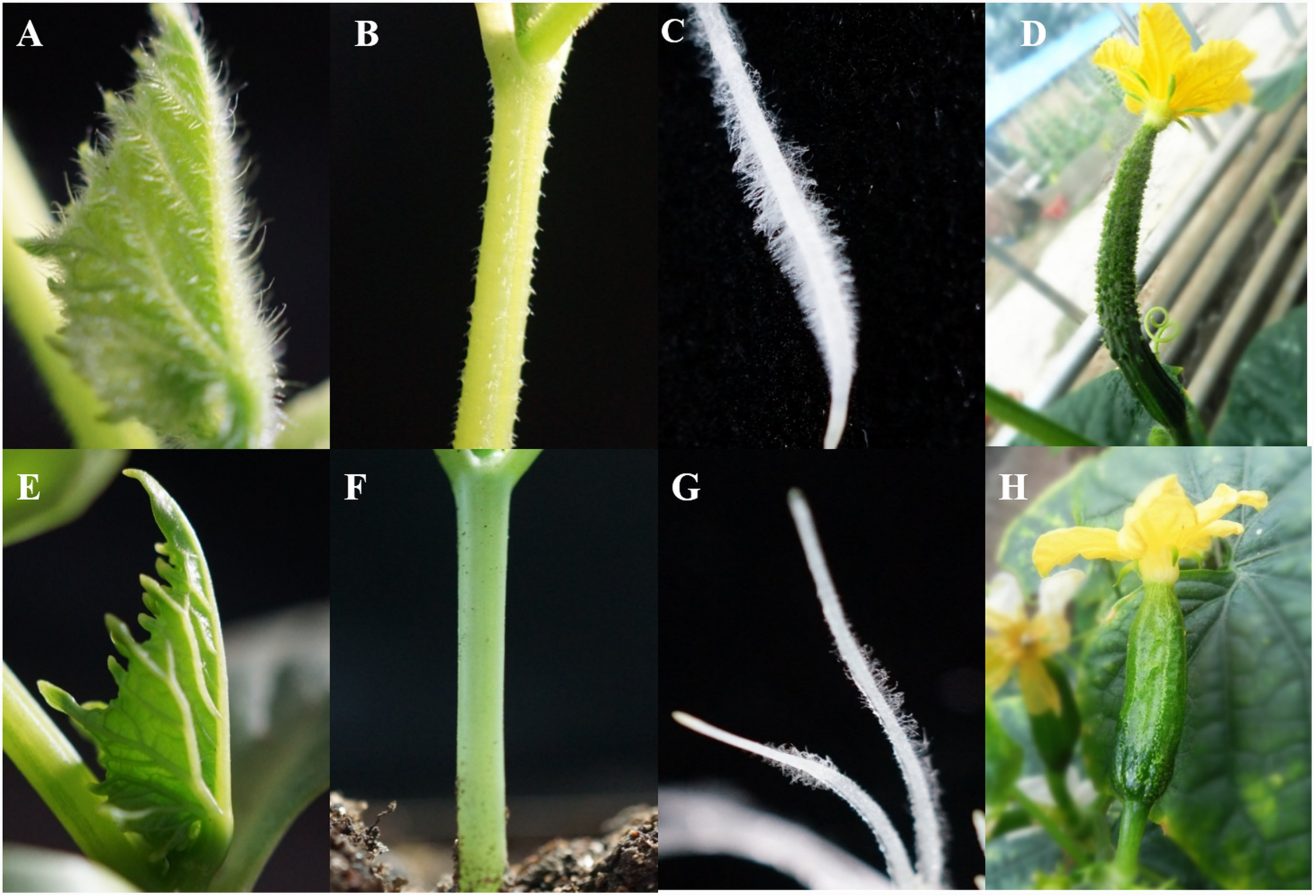
Morphological characterization of leaf, stem, root, fruit in 9930 and NCG157. Leaf, stem, root, fruit of 9930 (A-D). Leaf, stem, root, fruit of NCG157 (E-H).

**Fig 2:**
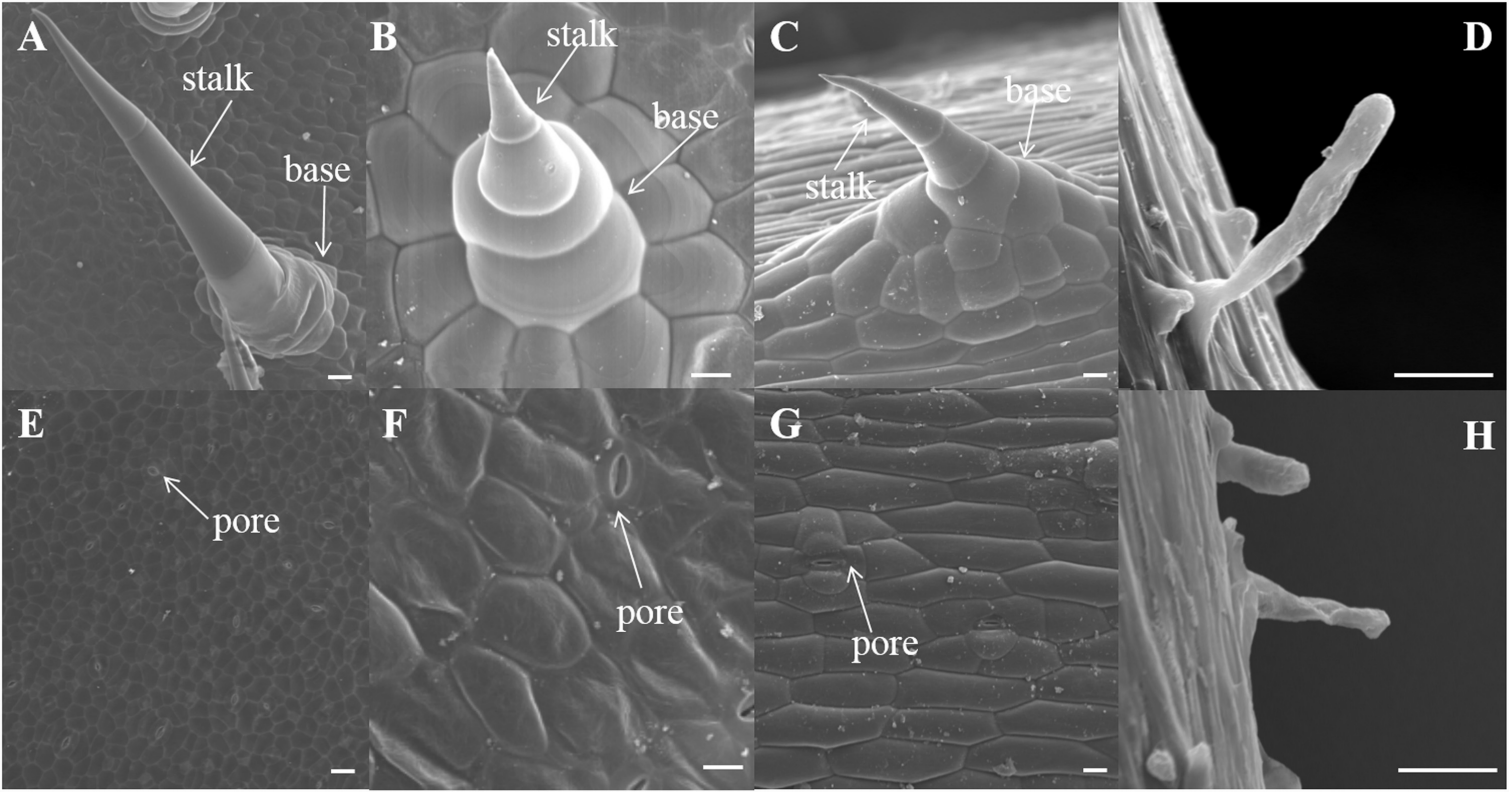
SEM images of leaf (A,E), cotyledon (B,F), stem (C,G) and root (D,H) in 9930 and NCG157. Bars, 100μm (A-H).

The trichomes present on the leaves, stems, roots and fruits of line 9930 (P_1_) are shown in Fig 1A–1D. The hairless foliage, smooth stem, root surfaces and glabrous fruit of the inbred line NCG157 (P_2_) are shown in Fig 1E–1H. The epidermal cells of 9930 were divided into either tuber-shaped trichomes or root hairs by SEM (Fig 2A–2D). Trichomes of leaf, cotyledon and stem (Fig 2A, 2B and 2C) were big, with a conical shape, and were non-glandular and branchless, and were of type II. The surface of NCG157 was smooth, except that the root was covered by root hairs (Fig 2E–2H).

### Inheritance of the glabrous trait in cucumber line NCG157

Three cross combinations were tested to examine the genetic relationships of three glabrous mutants (1945, NCG042 and NCG157) (S1 Table). By reciprocally crossing the three glabrous mutants, we obtained all F_1_ plants with trichomes. In the F_2_ populations, Chi-square tests were consistent with ratios of 9:7 (hairy:glabrous). So interactive multiple genes control the hairy trait, and also that was a new gene related to trichome production, different from *csgl1* and *csgl2*. We proposed this gene was designated as *csgl3*.

The genetic analyses of the cross between 9930 and NCG157 are shown in Table 1. In the F_2_, BC_1_P_1_ and BC_1_P_2_ populations, Chi-square tests were consistent with ratios of 3:1 (hairy:glabrous), 1:0 (hairy:glabrous), and 1:1 (hairy:glabrous), respectively. These results show that the hairy trait is controlled by a single dominant gene, putatively defined as *csgl3* gene.

**Table 1:** Segregation of hairy and glabrous plants among different populations derived from hairy cucumber line 9930 and glabrous cucumber NCG157.

Materials	#Lines/plants tested	H:G:U observed	H:G ratio tested	*χ*^*2*^	*P*
**9930 (P**_**1**_**)**	20		1:0	-	
**NCG157 (P**_**2**_**)**	20		0:1	-	
**F**_**1**_	20		1:0	-	
**F**_**1**_**’**	20		1:0	-	
**F**_**2**_	240	175:65:0	3:1	0.56	0.46
**F**_**2**_	1500	1153:347:0	3:1	2.79	0.10
**BC**_**1**_**P**_**1**_	120	120:0:0	1:0	-	
**BC**_**1**_**P**_**2**_	120	55:65:0	1:1	0.83	0.36

*H* hairy, *G* glabrous, *U* unassigned. ^a^Unassigned(*U*) plants were excluded from the calculation.

### Construction of SSR linkage groups and preliminary chromosomal mapping for the putative *csgl3* gene

**Fig 3:**
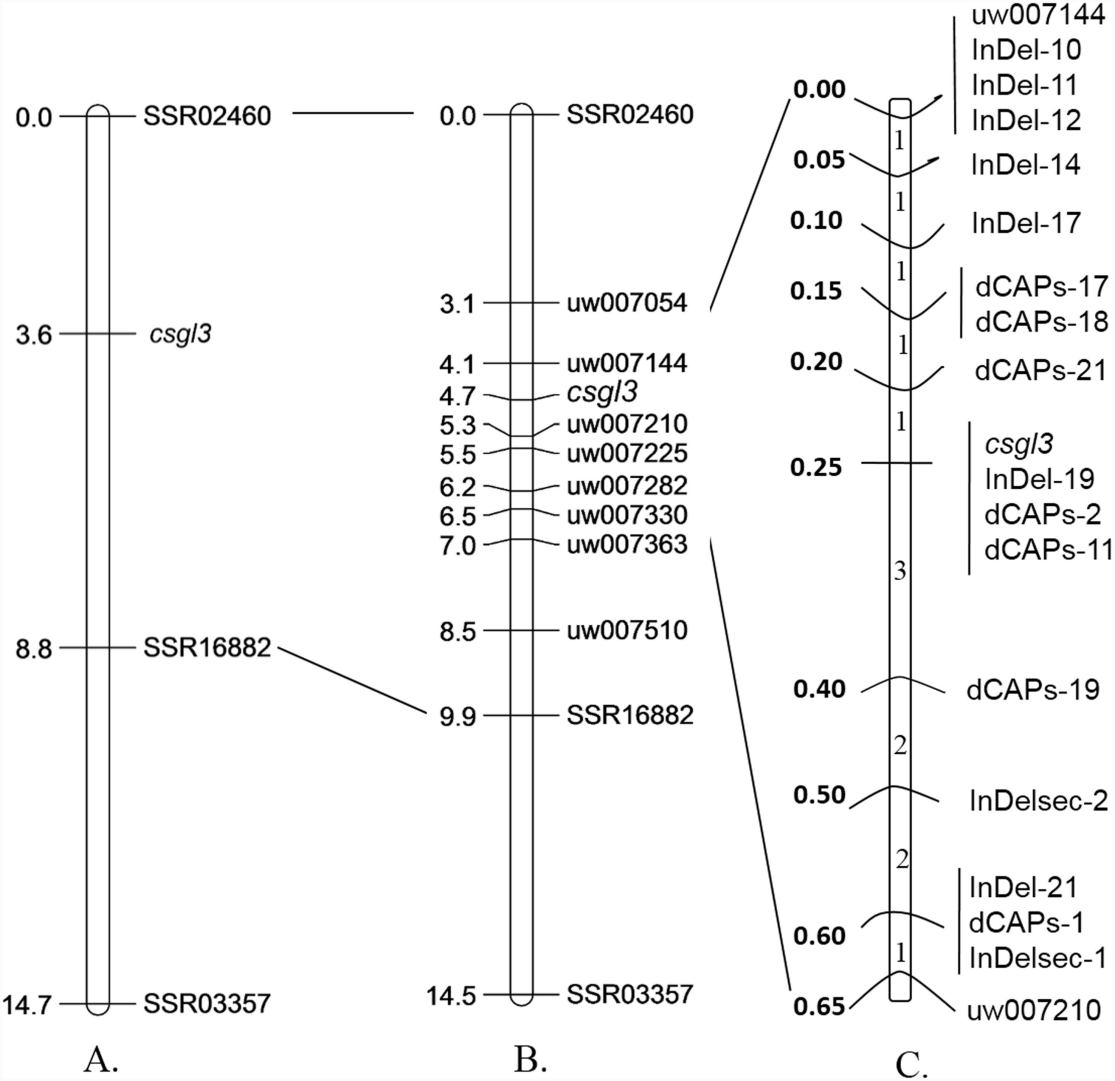
Fine mapping of the putative *csgl3* gene in cucumber. Map distance is given in centimorgans (cM).

Map-based cloning was carried out on the F_2_ population obtained from the cross of 9930 (P_1_) and NCG157 (P_2_). 490 primer combinations from a total of 2,112 (23.2%) generated polymorphic bands between P_1_ and P_2_. These markers were used to examine a possible linkage relationship with the putative *csgl3* gene using the DNA pools prepared from hairy wild-type phenotype and glabrous mutants. When 240 F_2_ plants were used in the linkage analysis, markers SSR03357, SSR16882 and SSR02460 were detected on chromosome 6. Finally, the region between SSR markers SSR02460 and SSR16882 was primarily mapped with genetic distances 3.6 cM and 5.2 cM, respectively (Fig 3A). 212 pairs of new UW SSR markers were used to examine the region between SSR02460 and SSR16882. Eight of these exhibited polymorphisms between the two parents, and were used for fine mapping. Based on 800 F_2_ individuals (randomly selected from 1500 F_2_ individuals), the new *csgl3* gene was mapped between UW007144 and UW007210, both with genetic distances of 0.6 cM (Fig 3B). These two markers were located in the same genome scaffold (scaffold000002) of line 9930, and the physical distance was approximately 125 kb.

### Fine mapping of the *csgl3* gene using new molecular markers

13 of the 1500 F_2_ plants showed evidence of recombination between UW007144 and UW007210. According to the sequence of line 9930 and the re-sequence data, several insertion/deletion locations and single-nucleotide polymorphisms (SNPs) were found between the parental lines using sequencing primers designed with the Primer Premier 6.0 program. To narrow down the search for the target gene *csgl3*, 42 InDel and 21 dCAPs markers were developed for fine mapping. 16 of these exhibited polymorphisms between the two parents, and then were used for linkage analysis on 13 recombinants plants (Fig 3C). No recombinant plants were detected with dCAPs-2, dCAPs-11 and InDel-19. It indicated that they co-segregated with *csgl3*. However, the *csgl3* gene was mapped between markers dCAPs-19 and dCAPs-21 with a linkage value of 0.2 cM and a physical distance of approximately 19.6 kb in scaffold000002 of 9930. All markers used for primary mapping and fine mapping are listed in Table 2.

**Table 2:** The sequences of SSR, InDel and dCAPs primers used to produce the genetic linkage map of the putative *csgl3* gene.

Marker type	Primer name	Forward primer	Reverse primer
**SSR markers**			
	UW007132	CCAAGTCCTTCCTGCATTTG	AGCAGCCATCGATTTCTCAG
	UW007144	GCAAGGGAAGGATCCTGTTA	CTTGCAGGAATTTTCACGGT
	UW007210	CGTGCGTGAATATCCACAAT	TCATTCCAAAATACACACACCTTT
	UW007225	ACAAGGTGGATTTGGAGCTG	TCGAGAGCCTCTTCACTGCT
	UW007228	GGTATCAAATTGAAAATTGGTAAAAA	ATGAGCCCATAAACGGGATT
**InDel markers**			
	InDelsec-1	GCTGTGTGTTGTAGTGATAC	AAATCTCAAATCGGCTTTCG
	InDelsec-2	CTGGTCATATTACTGGAGCTA	GAGAGTTGTATCGTAGAATGC
	InDel-11	TCGATCCCCAGGTATGACTC	GGATATCCATTAAACTACCCCAGA
	InDel-12	AACTCAGCATCCCACAAAGC	CCATGGTTGGACCTGTGATA
	InDel-14	CAATGCAAAAGGATTCAACG	AGGCCCCTTTCTGTTTATGG
	InDel-17	AGTTGATTGGTTTGGGTAATGA	GAATGAGTACAACGCCGTGA
	InDel-18	TTCAACCTTGTCTTGGCTTTT	AGTCAGCAACCTTGCCTTTG
	InDel-19	CATTTGACCAAACGCACACT	TGTCACAGCACAATGGACCT
	InDel-20	TCCTCATTTAATTTGGTTGTCAAA	CCCGAGAAAAGGATCAAACA
**dCAPs markers**			
	dCAPs-1	AGAAATAAAACAAAACAGATGAT	CCTTGGGAATTAGTAGACCAATGA
	dCAPs-2	AGGGGAAATGTATGGAGCAGCAAA	TCCGCCTCATTAAGAACCAC
	dCAPs-11	TAATCCTCCCAGTTGATAATAAC	TAAAGTCAATGCCCGAACCT
	dCAPs-17	GAATGTTGGGCCGAGTTGGTCT	GATTTAGGCCTTTGCGATGA
	dCAPs-18	TTTTTTTATATCTTTTTTCTTATG	TTTCATGTTTGTTTACGTTTAGTCTG
	dCAPs-19	GTAGGGTGCCACGTGGTGCTAAGAG	TTTGATTACTTTGGGAATTAGGG
	dCAPs-21	GTAAACACGGTAGAGTCTCCAT	CGACCTTCCACGTAATGGAT

### Annotation and genes prediction in the genomic region harboring the *csgl3* gene

The FGENESH program (Softberry, Inc., Mount Kisco, NY, USA) was used on the 19.6 kb genomic DNA region in scafflold000002 of the 9930 draft genome delimited by dCAPs-19 and dCAPs-21. Two genes, *Csa6M514860* and *Csa6M514870*, were predicted, and these could be inferred also from published data in the Cucurbit Genomics database (http://www.icugi.org) for this region.

**Fig 4:**
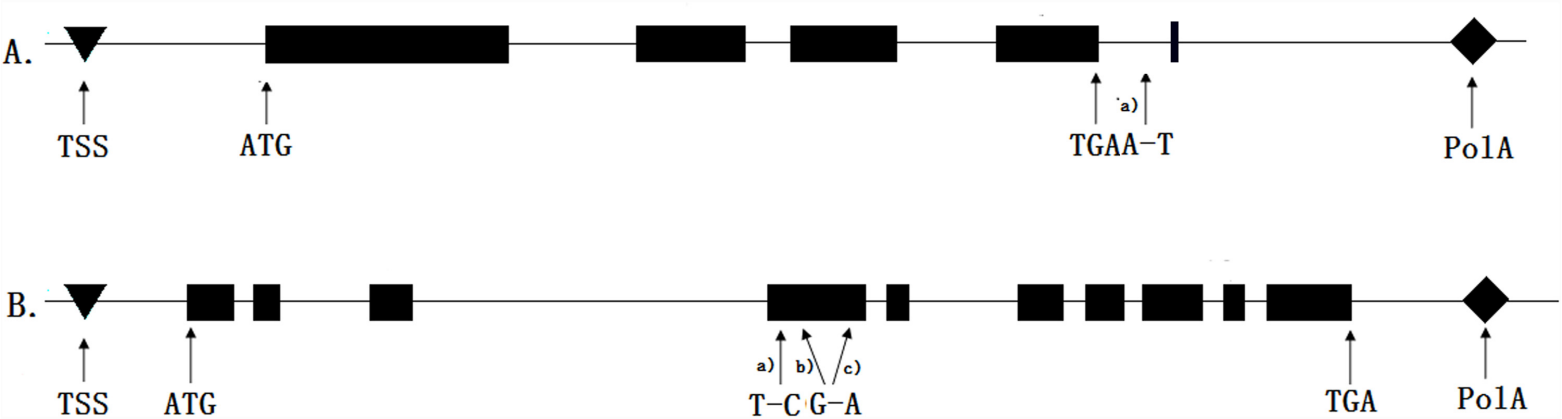
Predicted genes structure and mutations in two parental lines. A. is the structure and mutations of *Csa6M514860*. B. is the structure and mutations of *Csa6M514870*.

The coding region of *Csa6M514860* is comprised of 5 exons and encodes a 391-amino acid protein (Fig 4A). Alignment of the *Csa6M514860* candidate gene sequences revealed that a single nucleotide mutation (A to T) occurred at sequence 2206bp in intron, and no amino difference between the two parental lines.

The coding region of *Csa6M514870* gene is comprised of 10 exons and 9 introns encodes a 721-amino acid protein (Fig 4B). Genomic sequence analysis revealed that *Csa6M514870* gene carries 3 single-nucleotide transition T→C (611bp), G→A (820bp/865bp) at the fourth exon (Fig 4B a,b,c). These three nucleotide transitions resulted in an amino acid change from “Glu to Lys”and “Gly to Ser”, respectively.

### The expression levels of candidate genes, *Csa6M514860* and *Csa6M514870*, in different tissues

**Fig 5:**
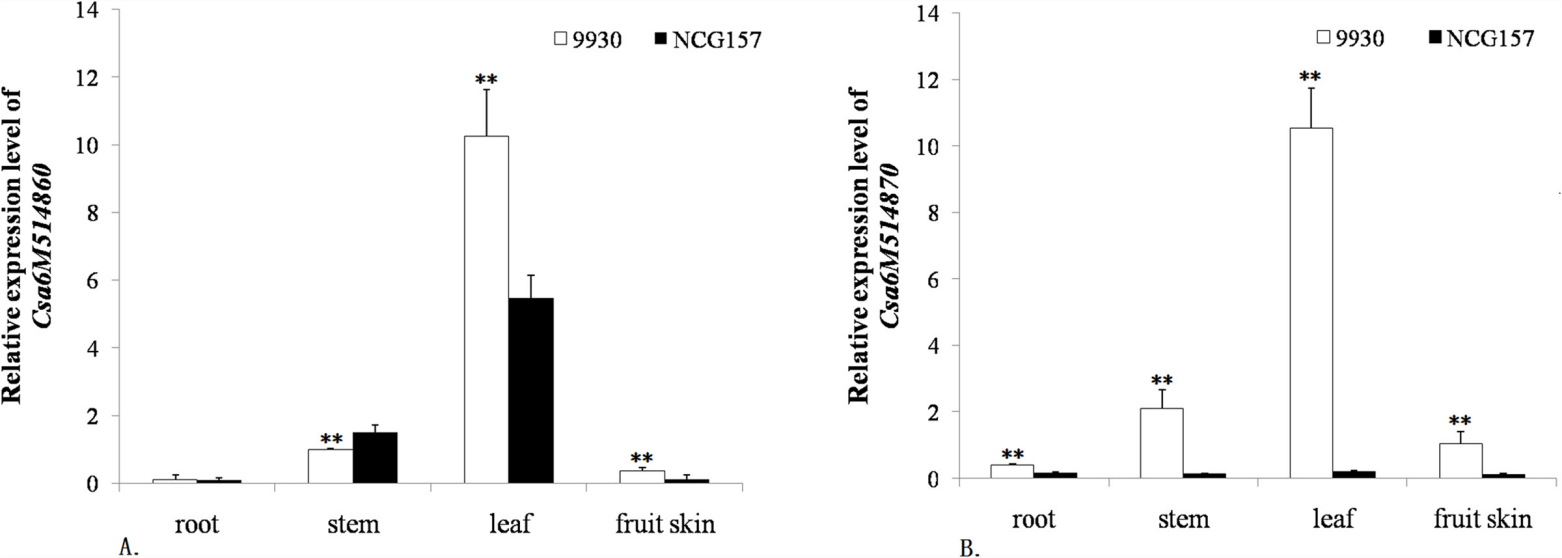
Relative expression levels of *Csa6M514860* (A) and *Csa6M514870* (B) in different tissues in 9930 and NCG157. Values are the mean ±SD (n = 3). (**indicates significant differences between 9930 and NCG157 at P = 0.01. Average in the graphs represents biological replicates).

The expression of *Csa6M514860* and *Csa6M514870* genes was investigated. Total RNA was extracted from root, stem, leaf and fruit skin, and qRT-PCR analyses were performed. *Csa6M514860* was expressed in all tissues of 9930 and NCG157, and was mainly expressed in leaves. In root, leaf and fruit skin, the expression of *Csa6M514860* in 9930 was higher than that of NCG157. But in stem, it showed opposite trend (Fig 5A). *Csa6M514870* was expressed in all tissues of 9930, and was highest in the leaves, but was only slightly expressed in NCG157 (Fig 5B). The expression was always higher in the wild-type parent compared to the glabrous mutant. These results indicated that the level of mRNA expression of *Csa6M514870* in different tissues was consistent, and the mutation in the coding region of 9930 could explain the variation in its mRNA expression. Primers used to the target genes and *Actin1* were listed in Table 3.

**Table 3:** The primer sequences of quantitative real-time PCR.

Primer name	Forward primer (5’-3’)	Reverse primer (5’-3’)
***Csa6M514860***	AAACCGATTTGGATTTCTGGA	TGCGACCTTGGACATGCTTA
***Csa6M514870***	GACGAGGAAGAGCACTGAC	ACGAAGCAAGGAGACACAAT
***Actin1***	TCCACGAGACTACCTACAACTC	GCTCATACGGTCAGCGAT

## Discussion

A number of glabrous mutations in plants are known but the mechanism of mutation and the responsible loci have not been understood at the molecular level. Trichome development in *Arabidopsis* has been intensively studied. The interplay of transcriptional regulators and hormone action has been shown to be involved in the developmental process [26]. In particular, the transcriptional activation of *GLABRA2* [27] by a regulatory complex formed from *GLABRA1* (*GL1*) [28], *TRANSPARENT TESTA GLABRA1* (*TTG1*) [16], and *GLABRA 3/ENHANCER OF GLABRA3* (*GL3/EGL3*) [29, 30] is a key function in the initiation of trichomes.

Cucumber spines are a highly valuable, external quality trait related to the market value of cucumbers [23]. In addition, glabrous cucumber plants exhibit resistance to aphids, and therefore, the use of pesticides can be reduced in the production of these types. Two mutant cucumber genes, *csgl1* and *csgl2*, have been found [14, 15]. Plants containing *csgl1* are spontaneous glabrous mutants from Northern China without any trichomes on their surface. Li et al. [14] used this mutant and a wild-type to isolate the *csgl1* gene by map-based cloning. This gene encodes a member of the HD-Zip I proteins which function in the abiotic stress responses of plants. A second mutant gene, *csgl2* occurs in the American processing type which exhibits very few trichomes on the plant surface. This was mapped to chromosome 2 [15].

In this work, a new cucumber glabrous mutant gene, *csgl3*, was isolated and characterized. Plants containing mutated gene have no trichomes on leaves, stems or fruit, and this feature is maintained throughout their life cycle. Based on the inheritance analysis of three glabrous mutants, all F_1_ plants were wild type, showing epidermal hair. These results are consistent with two complementary interacting genes controlling the hairy trait, and also that *csgl3* is a novel trichome development mutant in cucumber with distinct characteristics.

Our study was the first to use over 2,000 molecular markers to map the *csgl3* gene in cucumber. We successfully achieved fine-mapping for the gene. It was delimited to a 19.6 kb genomic region on chromosome 6 with two predicted candidate genes. *Csa6M514860* encodes the glucose-6-phosphate translocator 2. It mediates the process of starch biosynthesis [31] and may be related to the biological process of pollen maturation [32]. Analysis of the sequence of *Csa6M514860* revealed that there were no mutant sites in the exon, and the results of the qRT-PCR analysis were not consistent with the phenotypic characters.

Three markers, InDel-19, dCAPs-2 and dCAPs-11, designed from the sequence of *Csa6M514870*, co-segregated with the trait. In addition, *Csa6M514870* was found to harbor 3 single-base substitutions: T→C (611bp), G→A (820bp), and G→A (865bp) at the fourth exon resulting in a change to the amino acid sequence. *Csa6M514870* encodes a transcription factor, belonging to the HD-Zip family. The HD-Zip protein contains a conserved homeobox (HD), and a leucine zipper domain (LZ) closely linked to the C-termimal of the HD [33]. The *Arabidopsis* HD-Zip IV family consists of 16 members, among which *PROTODERMAL FACTOR2* (*PDF2*) plays an indispensable role in the differentiation of shoot epidermal cells as a transcription factor [34,35]. The sequences of *Csa6M514870* and *PDF2* are closely aligned. *PDF2* encodes a homeodomain protein that is expressed in the *L1* layer of the vegetative, floral and inflorescence meristems, and it also binds to the *L1* box promoter element which is required in some proteins for *L1* specific expression [36].

The expression level of *Csa6M514870* in the stem, leaf and skin of the fruit of the wild-type 9930 was higher than that in the mutant plants (Fig 5B), which was consistent with their phenotypic characters. The expression of *Csa6M514870* in the root tissue was also higher in 9930 than in NCG157. Therefore, it is reasonable to postulate that *Csa6M514870* is the candidate gene for the development of trichomes in cucumber. However, further evidence is needed to functionally validate this.

This research will lead to the development of a marker for trichome development in hybrid cucumber production and facilitate marker-assisted selection (MAS) of the smooth plant trait in cucumber breeding. It will also lead to an understanding of the way in which trichomes develop and the future cloning of the *csgl3* gene.

## Supporting Information

S1 Table Segregation of hairy and glabrous plants among different populations derived from three glabrous mutants. (DOCX)
