# Supplementary material for: A New Glabrous Gene (csgl3) Identified in Trichome Development in Cucumber (Cucumis sativus L.)
Source: PLoS One. 2016 Feb 4;11(2):e0148422. doi: 10.1371/journal.pone.0148422 (PMC4741392; doi:10.1371/journal.pone.0148422)
Supplement: S1 Table — (DOCX) [file pone.0148422.s001.docx]

**S1** **Table** Segregation of hairy and glabrous plants among different populations derived from three glabrous mutants.

| Cross lines | Materials | Plants tested | H:G:U observed | H:G ratio tested | *χ^2^* | *P* |
| --- | --- | --- | --- | --- | --- | --- |
| 1945×NCG042 | P_1_(1945) | 20 | 0:20:0 | - | - | - |
|  | P_2_(NCG042) | 20 | 0:20:0 | - | - | - |
|  | F_1_ | 20 | 20:0:0 | - | - | - |
|  | F_1_’ | 20 | 20:0:0 | - | - | - |
|  | F_2_ | 247 | 146:95:6 | 9:7 | 1.84 | 0.18 |
|  | BC_1_P_1_ | 105 | 69:33:3 | 1:1 | 12.7 | 0.00 |
|  | BC_1_P_2_ | 106 | 48:57:1 | 1:1 | 0.77 | 0.38 |
| 1945×NCG157 | P_1_(1945) | 20 | 0:20:0 | - | - | - |
|  | P_2_(NCG157) | 20 | 0:20:0 | - | - | - |
|  | F_1_ | 20 | 20:0:0 | - | - | - |
|  | F_1_’ | 20 | 20:0:0 | - | - | - |
|  | F_2_ | 271 | 167:104:0 | 9:7 | 3.18 | 0.08 |
|  | BC_1_P_1_ | 106 | 50:50:6 | 1:1 | 0 | 1.00 |
|  | BC_1_P_2_ | 105 | 50:52:3 | 1:1 | 0.04 | 0.84 |
| NCG042×NCG157 | P_1_(NCG042) | 20 | 0:20:0 | - | - | - |
|  | P_2_(NCG157) | 20 | 0:20:0 | - | - | - |
|  | F_1_ | 20 | 20:0:0 | - | - | - |
|  | F_1_’ | 20 | 20:0:0 | - | - | - |
|  | F_2_ | 265 | 153:112:0 | 9:7 | 0.24 | 0.63 |
|  | BC_1_P_1_ | 107 | 48:59:0 | 1:1 | 1.13 | 0.29 |
|  | BC_1_P_2_ | 106 | 42:64:0 | 1:1 | 4.57 | 0.03 |
| \| *H* hairy, *G* glabrous, *U* unassigned \| \| \| --- \| --- \| \| ^a^Unassigned(*U*) plants were excluded from the calculation \| | | | | | | |
